# Metagenomic analysis and identification of emerging pathogens in blood from healthy donors

**DOI:** 10.1038/s41598-020-72808-8

**Published:** 2020-09-25

**Authors:** Min Xu, Jing Gao, Shilin Li, Min Zeng, Jianming Wu, Mao Luo

**Affiliations:** 1 Institute of Blood Transfusion, Chinese Academy of Medical Sciences and Peking Union Medical College, Chengdu, 610052 China; 2 Collaborative Innovation Center for Prevention and Treatment of Cardiovascular Disease of Sichuan Province, Drug Discovery Research Center, Southwest Medical University, 319 Zhongshan Road, Luzhou, 646000 Sichuan China; 3 Department of Pharmacy, The Affiliated Hospital of Southwest Medical University, Luzhou, Sichuan China; 4 Laboratory of Chinese Materia Medica, Department of Pharmacology, School of Pharmacy, Southwest Medical University, Luzhou, 646000 China

**Keywords:** Metagenomics, Pathogens, Virology

## Abstract

Emerging infectious pathogens that threaten blood transfusions are known to be present in blood samples from healthy/qualified donors. The objective of this study was to investigate the microbiome of blood from healthy donors from the Luzhou area in southwestern China. Potential pathogens and cytomegalovirus (CMV) infection in the donor blood were identified. Total plasma nucleic acids were extracted from one pool of 5734 samples and were constructed for metagenomics analysis using Illumina sequencing. The microbiome and potential emerging/re-emerging pathogens were identified using bioinformatics analysis. Moreover, CMV antigen was measured via an enzyme-linked immunosorbent assay, and the CMV DNA level was assessed by quantitative RT-PCR. A total of 132 bacterial reads, 65 viral reads and 165 parasitic reads were obtained. The most frequent bacterium was *Escherichia coli* (95/132, 72%) with 95 reads in 132 bacterial reads, and the most prevalent parasite was *Toxoplasma gondii* (131/165, 79%). Among the viruses, *cytomegalovirus* (44/65, 68%) accounted for the highest frequency*,* followed by *Hepatitis E Virus* (10/65, 15%). Moreover, the positive rate of CMV-IgG was 46.25% (2652/5734), and the positive rate of CMV-IgM was 5.82% (334/5734). The positive rate of dual positive (IgG+ and IgM+) CMV was 0.07% (4/5734). Twenty-one (0.37%) specimens from 5734 donated blood samples were positive for CMV DNA. The CMV DNA levels ranged from 7.56 × 10^2^ to 3.58 × 10^3^ copies/mL. The current study elucidated the microbiome structure in blood from healthy/qualified donors in the Luzhou area and identified emerging/re-emerging pathogens. This preliminary study contributes to information regarding blood transfusion safety in China.

## Introduction

Blood is frequently used to treat life-threatening injuries and diseases but can also disseminate a large number of diseases^1–3^. Healthy/qualified blood donors contain many emerging infectious pathogens that could be transmitted through a blood transfusion or plasma derivative usage^4–6^. The residual pathogenic factors in screened blood consist mainly of emerging and re-emerging pathogens, new mutations in known pathogens, asymptomatic infection activation and expansion of the distribution areas of existing pathogens. Although developed countries in Europe and the Americas have already launched routine blood screening in epidemic areas or during epidemic seasons of some emerging pathogens^1,2,7^, epidemiological surveys of these undetected pathogens in blood donors have not been implemented in China.

Recently, emerging/re-emerging infectious diseases have become important threats to human life and safety^1,2,8^. More than 12% of human infectious diseases are categorized as emerging/re-emerging infectious diseases^9^. A wide range of emerging/re-emerging infectious pathogens exist, the classification of which is complicated by the high degree of genetic variation. Therefore, routine analytical methods cannot completely detect and identify emerging/re-emerging pathogens^8,10^. Human cytomegalovirus (HCMV, also known as human herpesvirus 5) is a ubiquitous β herpes virus^11,12^ that frequently causes various infections in individuals. Recent studies have shown that HCMV infections can be transmitted by blood transfusion^13,14^, which leads to a primary infection and the propagation of re-infection^15,16^. The present study provides important preliminary information on HCMV in populations of heathy blood donors in the Luzhou area.

To assess the current epidemiology of pathogens in the Luzhou area in southwest China, increase awareness of potential pathogens and ensure a safer blood supply, we performed metagenomics analyses using Illumina HiSeq high-throughput sequencing to detect the microbiome and identify emerging/re-emerging pathogens in blood samples from healthy/qualified and screened blood donors in the Luzhou area. Furthermore, the CMV levels and CMV DNA were measured via an enzyme-linked immunosorbent assay (ELISA) and quantitative real-time PCR system, respectively.

## Materials and methods

### Sample preparation and library construction

All study subjects were Han Chinese individuals who voluntarily participated and signed an informed consent form. The study was conducted in accordance with the Declaration of Helsinki and was approved by the Human Ethics Committees of the Luzhou area Blood Center (blood bank), Affiliated Hospital of Southwest Medical University, and Chinese Academy of Medical Sciences & Peking Union Medical College, Sichuan province, P. R. China. More than 5734 samples from healthy/qualified blood donors from the Luzhou area Blood Center (blood bank) were randomly selected from August 2017 to August 2018. All blood samples were grouped and stored at − 20 °C until further analysis. The pooled samples consisted of 10 µL of blood from each of the 5734 donors, which were then grouped based on biological replicates. Three technical replicates were also used for each pooled sample. To minimize pathogen loss, the extracted DNA was quantified using a Nanodrop and had a concentration of at least 50 ng/μL. The total nucleic acid amount was at least 1.6 μg. Human mitochondria were removed by centrifugation at 3000 × *g* for 10 min and filtration through 0.45-μm and 0.22-μm filter membranes. Each sample was ultracentrifuged at 41,000 rpm for 120 min, and the supernatant was removed. The resulting pellet was resuspended in 450 μL of PBS, and free DNA was digested using DNase I. Next, the suspended DNA and RNA were extracted using the High Pure Viral Nucleic Acid Large Volume Kit (Roche). RNA was reverse transcribed into cDNA using the Transcriptor First Strand cDNA Synthesis Kit (Roche). The concentration and total amount of the cDNA libraries constructed were greater than or equal to 50 ng/μL and 1.6 μg, respectively.

### Illumina HiSeq 4000 sequencing and bioinformatics analysis

The cDNA libraries were sent to Novogene (Tianjing, China) for high-throughput sequencing using the Illumina HiSeq 4000 platform. The samples were used to construct the PE150 library, and upstream quality control (QC) of the raw data was completed. The bioinformatics analysis consisted of 3 main steps. First, the adaptor sequences were deleted. Second, very low-quality reads were removed. If a read had over 50% bases with Q ≤ 5, it was considered a low-quality read and removed. Third, duplicate reads were removed. Finally, the sequences with Q30 > 70% were identified using MCS 2.0 software, resulting in approximately 2 GB of data. The raw data contained a large amount of nontarget sequences, which were mainly from parasites (human). Therefore, data filtration was necessary before further processing to remove the human sequences. Then, all raw data were compared to the human genome using Bowtie2 software, which is a large-scale comparison software program developed specifically for second-generation sequencing with high efficiency, speed and accuracy. Matched reads that represented data from humans and were nontarget sequences were filtered. A sensitive model was selected as the basic parameter and the others were used as defaults. After filtration, the data were applied for Blastn, Blastx and tBlastx sequence comparisons. Sequences with E > 10–3 were considered nonidentifiable. Because the input sequences were shorter, most of the data yielded results with smaller E-values.

Filtered sequencing read mapping to reference genomes was performed using the Burrows–Wheeler Aligner (BWA) alignment software that performs fast alignments of short sequences against a reference sequence. Specifically, if all results in the match set belonged to one species, then they belonged to that species. Moreover, if they belonged to a different species in a single genus, they belonged to that genus, and if they belonged to different genera in the same family, they belonged to the same family. Based on this logic, all results underwent taxonomy allocation. Once all the results were obtained, the total species and dominant species of the microbiome in each sample could be statistically analyzed.

### Detection of immunoglobulin G (IgG) and IgM antibodies to CMV with a commercial ELISA kit

The CMV levels in the 5734 blood samples were measured via ELISA. The CMV antibodies in specimen serum were detected using an anti-CMV IgG/IgM ELISA kit following the manufacturer’s protocol (Human anti-cytomegalovirus antibody IgG ELISA Kit,) and Human anti-cytomegalovirus antibody IgM ELISA Kit, Cusabio, USA). The selected ELISA reactive samples were used as external controls on the first and last plate during each testing day as an additional QC measure. A positive result (S/C.O. ≥ 1) was considered for samples that had an absorbance greater than or equal to the cut-off value, which indicated the presence of CMV antibodies.

### CMV DNA detection by real-time PCR assay

DNA was extracted from 200 μL of each serum sample using the QIAamp DNA Blood Mini Kit (Qiagen). The DNA extracts were stored at − 80 °C before PCR analysis. All the ELISA-positive samples were tested for CMV (AY186194.1). Real-time PCR was used to detect CMV DNA in the plasma samples. Standard curves were generated using the quantified DNA containing the targeted sequences in the CMV major immediate-early (MIE) gene by inserting 136-bp conserved region fragments into a PTA2 Vector. All RT-PCRs were performed on an ABI 7500 instrument (Applied Biosystems, Foster City, CA, USA) with 25 μL of the FastStart Universal SYBR Green Master (Rox) Kit (Roche) and 5 μL of DNA template. The primers used for the detection of CMV, Q-CMV-F (forward primer 5′-GACTATCCCTCTG TCCTCAGTA-3′) and Q-CMV-R (reverse primer 5′-AGACACTGGCTCAGACTTGA-3′), were used to amplify a 136-bp segment from the MIE gene. Negative controls that used water as a template and positive controls that used 500 plasmid copies as a template were also included in each run. The cycling conditions were as follows: 1 cycle of 95 °C for 10 min; 45 cycles of 95 °C for 15 s, 58 °C for 30 s and 72 °C for 30 s; and a final cycle of 95 °C for 15 s, 60 °C for 15 s and a gradual increase to 95 °C for 30 min at a ramp rate of 2% to obtain the melting curves. The linear range and lower limit of 95% detection (95% LLOD) for the target was determined as recommended. The linear range of the CMV DNA assay extended from 10^8^ to 10^2^ copies/mL.

### Ethics approval

All human subjects used in the study have been reviewed by the Research Ethics Committee of the Luzhou area Blood Center (blood bank) and Affiliated Hospital of Southwest Medical University, Luzhou, and the Chinese Academy of Medical Sciences & Peking Union Medical College, Chengdu, Sichuan province, P. R. China, and have been performed in accordance with the 1964 Helsinki declaration and its later amendments or comparable ethical standards. All samples were collected with informed consent of all subjects. There is no security and privacy violation to the patient’s health in our study.

### Informed consent

Informed consent was obtained from all individual participants included in the study.

## Results

### High-throughput sequencing results

**Figure 1:**
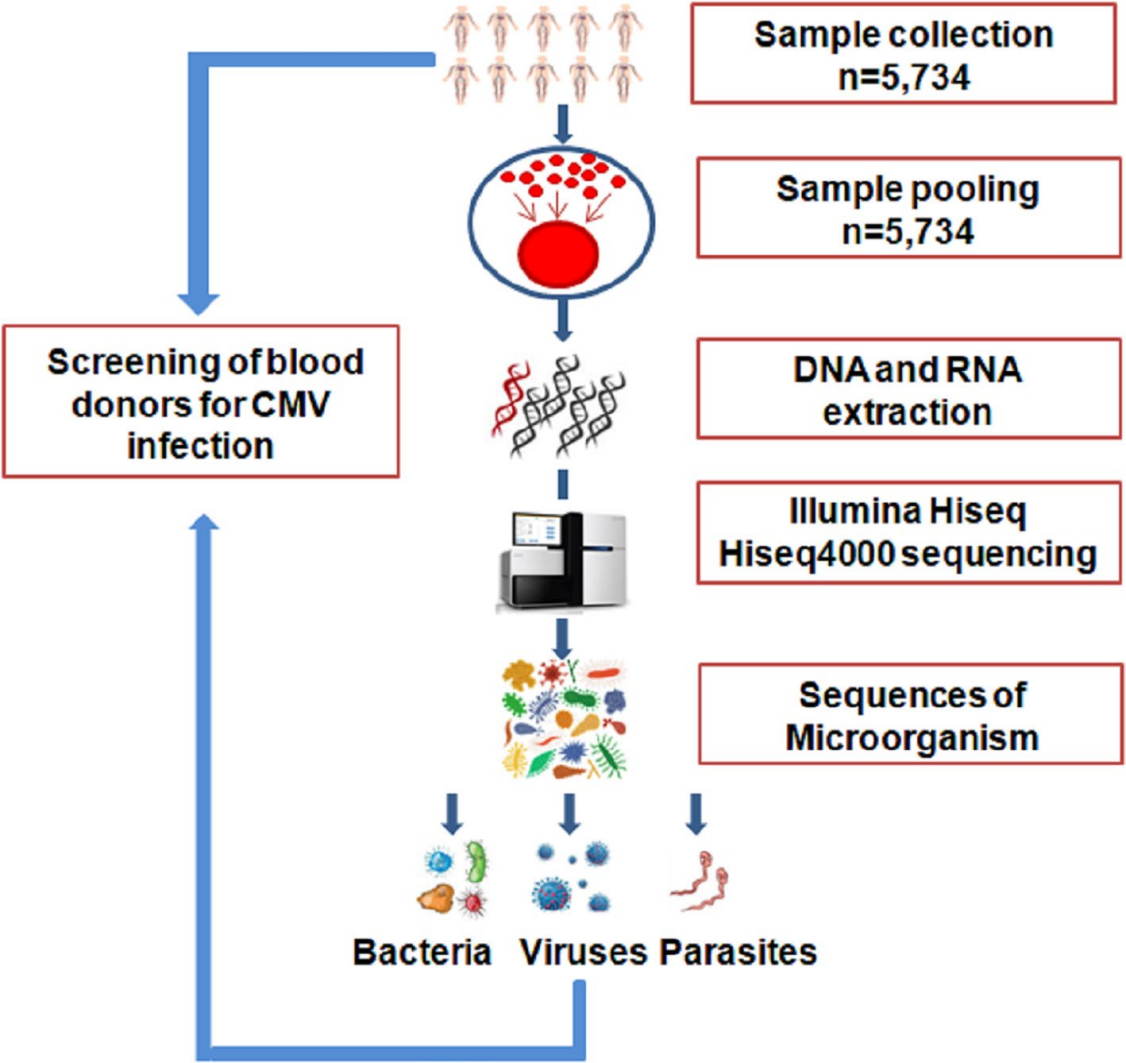
Schematic workflow representation. Schematic representation of the workflow for blood sample preparation, genomic extraction and metagenomics analysis using Illumina HiSeq 4000 high-throughput sequencing. CMV antibodies and CMV DNA were analyzed via ELISA and quantitative real-time PCR, respectively.

**Figure 2:**
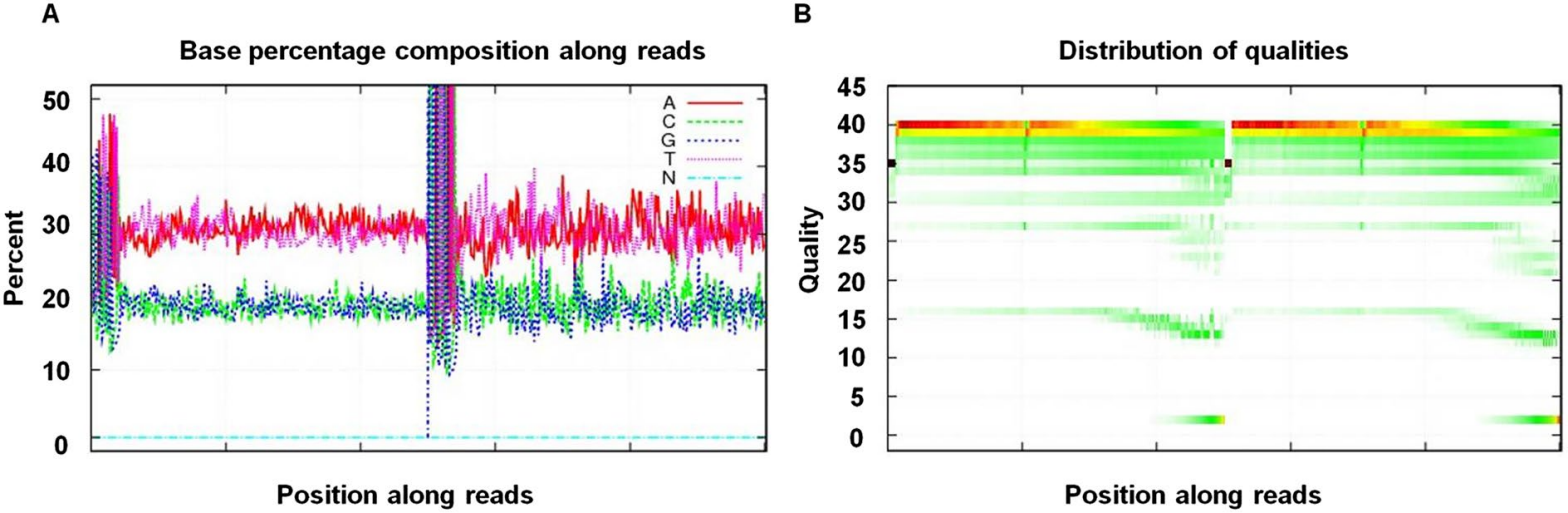
The base percentage distribution and read quality after data filtering. (**A**) shows the base percentage distribution along the reads after filtering; (**B**) shows the distribution of qualities along the reads after filtering.

After extraction, nucleic concentrations were quantified using a UV spectrophotometer (DNA/cDNA concentration should be 200 ng/µl). The sample libraries were sent to Novogene (Tianjing, China) for Illumina HiSeq 4000 high-throughput sequencing to obtain raw data. The workflow is shown in Fig. 1. The raw data from the HiSeq were deposited in the short reads archive of GenBank. The base percentage distribution and read qualities in data filtering are shown in Fig. 2A, B, respectively.

The adaptor sequences, contamination and low-quality reads were removed from the raw reads. The results are shown in Table 1. A total of 1.38 GB of DNA data were obtained, including 2,967,242 clean reads. Synchronously, 2.08 GB of cDNA data was obtained, including 3,450,046 clean reads (Table 1). The DNA pool generated 2,967,242 clean reads. The Q30 value, which indicates the percentage of bases with quality values larger than or equal to 30 (0.1% base error recognition rate), was 99.15%. Moreover, 3,450,046 clean reads were in the cDNA pool with a Q30 value of 98.83%. These sequencing results were both highly accurate and reliable (Table 1).

**Table 1:** Statistical results of MiSeq Illumina deep sequencing. Clean bases: total number of clean bases; Q20 (%)/Q30 (%): The percentage of nucleotides with a quality higher than 20 or 30/nucleotide (clean read1, read2); GC (%): Percent GC/nucleotide (clean read, read2).

Sample	Clean reads	Clean bases	Strategy	Q20 (%)	Q30 (%)	GC (%)
DNA pool	1,375,023,000	2,967,242	PE150	99.48	99.15	40.9
cDNA pool	2,082,710,000	3,450,046	PE150	99.30	98.83	39.87

### Sequence analysis of potential pathogens in the blood samples

**Figure 3:**
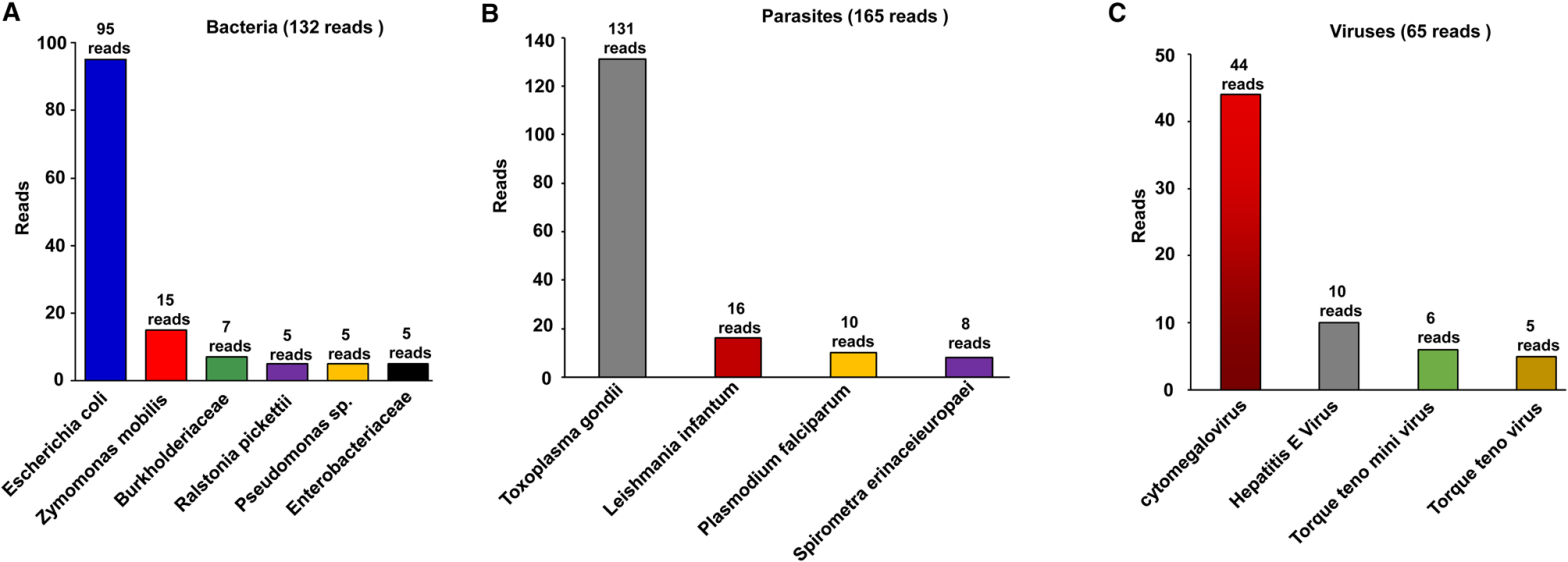
The number of pathogens of microbial community. The number of pathogens of microbial community from 5734 samples in blood from healthy donors were elucidated via bioinformatics analysis. (**A**) shows the result of bacteria. (**B**) shows the result of parasites. (**C**) shows the result of viruses.

To evaluate the microbial community and potential pathogens, an additional bioinformatics analysis was employed as described in Materials and Methods. The microbial community results show that 36.5% (132/362) of the sequences were from bacteria, followed by 18% (65/362) from viruses and 45.5% (165/362) from parasites, as shown in Fig. 3A–C and Table 2. Potential pathogens that had less than 5 reads were removed. Table 2 shows taxonomic categories of 132 reads from bacteria, 65 reads from viruses and 165 reads from parasites. Among the bacteria, the most frequent species were *Escherichia coli* (72%) with 95 reads, followed by *Zymomonas mobilis* (11%) with 15 reads, Burkholderiaceae (5%) with 7 reads and *Ralstonia pickettii* (4%), *Pseudomonas* sp. (4%) and *Enterobacteriaceae* (4%), each with 5 reads (Fig. 3A and Table 2). Among the parasites, *Toxoplasma gondii* (79%) accounted for the highest frequency with 131 reads, followed by *Leishmania infantum* (10%) with 16 reads and *Plasmodium falciparum* (6%) and *Spirometra erinaceieuropaei* (5%) with 10 and 8 reads, respectively (Fig. 3B and Table 2). Among the viruses, *cytomegalovirus* (68%) with 44 reads accounted for the highest frequency*,* followed by *Hepatitis E Virus* (15%) with 10 reads. Moreover, 2 viruses in *Anelloviridae* were detected, including *Torque teno mini virus* (9% with 6 reads) and *Torque teno virus* (8% with 5 reads) (Fig. 3C and Table 2).

**Table 2:** Annotation statistics of potential blood sample pathogens. After high-throughput sequencing and data filtering, bioinformatics analysis was employed to evaluate the microbial community and potential pathogens. The results show that 32.5% of sequences were obtained from bacteria, 15.1% from viruses and 2.5% from parasites.

	Potential pathogens	Reads		Potential pathogens	Reads
Virus (65)	*Cytomegalovirus*	44	Bacteria (132)	*Escherichia coli*	95
	*Hepatitis E Virus*	10		*Zymomonas mobilis*	15
	*Torque teno mini virus*	6		*Burkholderiaceae*	7
	*Torque teno virus*	5		*Ralstonia pickettii*	5
				*Pseudomonas* sp.	5
Parasite (165)	*Toxoplasma gondii*	131		*Enterobacteriaceae*	5
	*Leishmania infantum*	16			
	*Plasmodium falciparum*	10			
	*Spirometra erinaceieuropaei*	8			

The reads are specific and not an indication of environmental contaminants.

After the next generation sequencing (NGS), a total of 1.98 Gb of data were obtained, including 3,967,242 paired-reads, and 1,983,621,000 bases (bp). All the sequences with Q30 > 70% were identified using the MCS2.0 software, resulting in approximately 2 GB of data. After remove human sequences, data were used for Blastn, Blastx, and tBlastx sequence comparisons with NCBI library.

### CMV antibody and CMV DNA detection by ELISA and real-time PCR

The CMV IgG and IgM antibodies in healthy/qualified blood donor samples were measured by ELISA. As shown in Table 3, a total of 5734 serum samples were collected and screened from for CMV antibodies, of which 2986 samples tested positive (IgG or IgM) with a rate of 52.08% (2986/5734). The positive rate of CMV-IgG was 46.25% (2652/5734) while the positive rate of CMV-IgM was 5.82% (334/5734). The positive rate of both CMV-IgG and CMV-IgM was 0.07% (4/5734). No significant differences in the positivity rates were detected among sex, age, residence, profession, or ethnicity. The positive rate of CMV-IgG was 78.91% in age 45–55 group, it’s higher than other age groups. These results highlight the urgent need to test for CMV antibodies in donor blood to ensure safety.

**Table 3:** Clinical characteristics of CMV-IgG+, CMV-IgM+ and CMV-NAT+ in blood from healthy donors. The IgG and IgM antibodies of CMV in the blood samples were measured by ELISA. CMV DNA was detected by quantitative real-time PCR. No significant differences in the positivity rates were detected among sex, age, residence, profession and ethnicity. Of the 2986 positive samples (IgG or IgM), the positive rate of CMV-IgG, CMV-IgM and both CMV-IgG and CMV-IgM was 46.25%, 5.82%, 0.07%, respectively. Decreased CMV DNA was noted in the positive specimens (7.56 × 10^2^ to 3.58 × 10^3^ copies/mL). The positive rate of CMV-IgG was 78.91% in age 45–55 group, it’s higher than other age groups.

Category	Columns	Total n	CMV-IgG+ n (%)	CMV-IgM+ n (%)	Total (+) IgG+ or IgM+ n (%)	IgG+ and IgM+ n (%)	Real-time PCR CMV-NAT+ n (%)
Gender	Male	3214	1215 (37.80)	244 (7.59)	1459 (45.40)	1 (0.03)	5 (0.16)
	Female	2520	1437 (57.02)	90 (3.57)	1527 (60.60)	3 (0.12)	16 (0.63)
	Total	5734	2652 (46.25)	334 (5.82)	2986 (52.08)	4 (0.07)	21 (0.37)
Age	18–24	1461	494 (33.81)	22 (1.51)	516 (35.32)	0	2 (0.14)
	25–34	1708	552 (32.32)	155 (9.07)	707 (41.39)	3 (0.18)	9 (0.53)
	35–44	1432	712 (49.72)	117 (8.17)	829 (57.89)	1 (0.07)	7 (0.49)
	45–55	1133	894 (78.91)	40 (3.53)	934 (82.44)	0	3 (0.26)
Marital status	Married	2927	1791 (61.19)	132 (4.51)	1823 (62.28)	2 (0.07)	12 (0.41)
	Unmarried	2604	719 (27.61)	168 (6.45)	1077 (41.36)	2 (0.08)	8 (0.30)
	Unknown	203	52 (25.62)	34 (16.75)	86 (42.36)	0	1 (0.49)
Education	Masters degree	893	402 (45.02)	45 (5.04)	487 (54.54)	0	2 (0.22)
	Undergraduate	1416	583 (41.17)	38 (2.68)	721 (50.92)	2 (0.14)	5 (0.35)
	College Diploma	1121	498 (44.42)	45 (12.93)	643 (57.36)	1 (0.09)	5 (0.45)
	Under high-school	1198	531 (44.32)	184 (15.36)	815 (68.03)	1 (0.09)	6 (0.50)
	Unknown	1106	638 (57.68)	22 (1.99)	880 (79.57)	0	3 (0.27)

In this blood donor population, 21 CMV DNA-reactive samples were found by real-time PCR, accounting for 0.37% (21/5734), and the positive rate of both CMV-IgG and -IgM was 0.07% (4/5734).

A quantitative real-time PCR system was used to detect CMV DNA in the plasma samples. As shown in Table 3, 21 CMV DNA-reactive samples were found, accounting for 0.37% of the total blood samples (21/5734). Interestingly, decreased CMV DNA was noted in the positive specimens (7.56 × 10^2^ to 3.58 × 10^3^ copies/mL).

## Discussion

The advantages of metagenomics technology in blood transfusion research include high efficiency and broad pathogen coverage. In recent years, metagenomics technology has been used to analyze inorganic environments, including the ocean^17^ and soil^18^, and has also proven remarkably useful in studies of pathogens carried by animals such as birds, bats, turkeys and sea turtles^19^. The results from such analyses have allowed for the description of the microbiomes of these animals^20,21^. Here, we employed Illumina HiSeq 4000 high-throughput sequencing for metagenomics analysis to resolve the microbiome in the blood of 5734 healthy/qualified donors collected from 2017 to 2018 in the Luzhou area in southwestern China. We identified the taxonomy of emerging/re-emerging pathogens and cytomegalovirus (CMV) infection using the bioinformatics analysis, ELISA and quantitative real-time PCR.

In this study, we assessed the microbiome structure and demonstrated that healthy/qualified blood donors in southwestern China might carry emerging/re-emerging pathogens, including low-level CMV infection. We also showed that *Toxoplasma gondii* was the most prevalent parasitic pathogen, followed by *Leishmania infantum* and *Plasmodium falciparum*. *Toxoplasma gondii* infection is typically silent and is most commonly transmitted by animals^22,23^. Close contact between humans and infected animals is one of the major transmission routes of *Toxoplasma gondii* infection^22–24^. Moreover, *Toxoplasma gondii* infection can be transmitted through blood transfusion. Populations with low and defective immune function are particularly susceptible to acquiring *Toxoplasma gondii* infection from blood transfusion and can suffer severe consequences^25,26^. The blood collection and supply system in China does not perform routine screening for toxoplasmosis: however, whether *Toxoplasma gondii* detection should be performed for certain blood recipient populations is worth consideration. Furthermore, DNA fragments of pathogens that are considered threats blood transfusion safety in Europe and America^8^, including as *P. falciparum* and *L. infantum,* were discovered in this study. Considering that malaria and *Leishmania* infection are currently resurging^27,28^ and that Luzhou and the surrounding region are within the endemic area, the blood collection and supply system should enhance their surveillance of these parasites in donated blood samples. Many types of bacteria were also identified in this study, including *Escherichia coli*, which accounted for the highest frequency. These bacteria can potentially cause chronic infection in the blood and bone marrow. Further contamination may occur due to improper disinfection during blood collection or experimental processes. Therefore, blood collection personnel should maintain high disinfection standards when manipulating blood samples.

Previous studies have shown a certain prevalence of CMV in Chinese blood donors^29^. Interestingly, we found that the viral load of CMV infection was lower (below 10^4^ copies/mL) in southwestern China. Although a high number of reads with CMV were detected in samples from the Luzhou area, the positive rate of both CMV-IgG and CMV-IgM was low, and the quantitative DNA levels ranged from 7.56 × 10^2^ to 3.58 × 10^3^ copies/mL. CMV infection, characterized by host immunosuppression, is most commonly transmitted through blood transfusion and causes an asymptomatic infection or mild flulike symptoms^30^. Clinical trials have found that primary CMV is typically silent in pregnant women, healthy children and adults^31^. Populations with low and defective immune function are particularly susceptible to acquiring CMV infection from blood transfusion and can suffer severe consequences. The blood collection and supply system in China does not perform routine screening for CMV^32^. The data from this study suggest that CMV detection should be considered for certain blood recipient populations. Future studies are required to isolate viruses on the 21 qPCR-positive CMV samples. Interestingly, the positive rate of CMV-IgG was 78.91% in age 45–55 group and that was higher than other age groups, we speculate that it may depend their habits or immunity.Other viruses were identified in this study, including *Hepatitis E Virus* and 2 types of viruses in *Anelloviridae*: *Torque teno mini virus* and *Torque teno virus*. *Anelloviridae* infection causes a broad range of clinical manifestations as well as asymptomatic infection in humans^33,34^. Currently, data on these viruses in China are scarce. The infection rate of *Anelloviridae* in healthy/qualified populations in countries such as Japan is close to 100%, and the infection rates in Great Britain and America are approximately 10%^33,35^. A high viral load of *Anelloviridae* infection has been shown to cause some clinical symptoms in humans^34,36^; however, whether these viruses can cause disease remains unclear.

This study identified pathogens in the microbiome of donated blood samples and discovered emerging pathogens that are already present in the blood supply. These pathogens therefore pose a risk yet are not being tested for in the blood supply. Because of constraints related to the number of collected samples and time, we were unable to perform a comprehensive analysis that is truly reflective of the prevalence of emerging/re-emerging pathogens in healthy/qualified blood donor samples in southwestern China. Our data suggest that parasites should be an area of focus for blood donors in the Luzhou area. These prospective results obtained using metagenomics provide references for the surveillance of certain pathogens. Large-scale epidemiological surveys targeting specific parasites should be performed to understand the actual prevalence of these parasites in blood from healthy/qualified donors.
